# Real-world outcomes for Chinese breast cancer patients with tumor location of central and nipple portion

**DOI:** 10.3389/fsurg.2022.993263

**Published:** 2022-10-03

**Authors:** Wei-Da Fu, Xiao-Hui Wang, Kang-Kang Lu, Yi-Qiao Lu, Jie-Yu Zhou, Qi-Di Huang, Gui-Long Guo

**Affiliations:** ^1^ Department of Breast Surgery, The First Affiliated Hospital of Wenzhou Medical University, Wenzhou, China; ^2^ Department of Breast / Thyroid Surgery, Jinhua Municipal Central Hospital, Jinhua, China

**Keywords:** breast cancer, tumors located in central and nipple portions, tumors in the breast peripheral quadrant, lymph nodes metastasis, prognosis

## Abstract

**Background:**

The association between tumor location and breast cancer prognosis has been controversial. We sought to explore the relationship between tumors located in central and nipple portion (TCNP) and Chinese breast cancer.

**Patients and methods:**

A total of 1,427 breast cancer patients were recruited. There were 328 cases of TCNP and 1,099 cases of tumors in the breast peripheral quadrant (TBPQ). The chi-square test was used to compare different variables between TCNP and TBPQ groups. A one-to-one propensity score matching (PSM) was applied to construct a matched sample consisting of pairs of TCNP and TBPQ groups. Kaplan–Meier curves were used for survival analysis of disease-free survival (DFS), breast cancer-specific survival (BCSS) and overall survival (OS). The Cox proportional hazards regression model was applied to identify prognostic risk factors.

**Results:**

The median follow-up time was 58 months. Compared to TBPQ, TCNP patients had significantly larger tumor size, more frequent metastasis to lymph nodes (LN) and more proportions of TNM stage II–III. DFS, OS and BCSS rates were markedly lower in the TCNP group as compared to the TBPQ group before and after PSM (all *p* < 0.05). Multivariate Cox analysis showed that TCNP was an independent prognostic factor for breast cancer. Subgroup analysis indicated that for breast molecular subtypes and TNM stage II-III breast cancer, TCNP were related to worse prognosis. Multivariate logistic regression revealed that TCNP was an independent contributing factor for LN metastasis.

**Conclusion:**

In Chinese breast cancer, compared to TBPQ, TCNP is associated with more LN metastasis and poorer prognosis.

## Introduction

Breast cancer is the most common malignant tumor in women, which threaten to women's health seriously. In recent years, the importance of active screening and early diagnosis have been well acknowledged in the field which significantly improved the prognosis of breast cancer (1). However, breast cancer is still one of the main causes of female death, especially in developing countries, even more than lung cancer (2). Therefore, it is critical to identify prognostic risk factors of breast cancer so proper treatment strategy can be timely adjusted.

Studies have shown that many clinicopathological factors affected the prognosis of breast cancer patients, such as age, tumor size, body mass index (BMI), TNM stage, histological grade, lymph node (LN) status, Ki67, hormone receptor and human epidermal growthfactor receptor 2 (HER2) expressions (3–6). In recent years, molecular typing and gene detection have gradually entered the clinical practice to guide the precise treatment of breast cancer and improve the prognosis of breast cancer patients (7–9). However, the prognosis of breast cancer patients with similar clinicopathological characteristics can be varied even though receiving the same treatment, indicating that there are still many other factors affecting the prognosis.

Breast cancer most commonly locates at the upper outer quadrant and rarely at the lower inner quadrant of the breast. Previous studies have confirmed that the location of the primary mass of breast cancer could affect the prognosis of patients (10–14). Typically, tumor located at the medial quadrant was associated with worse prognosis, and the inner lower quadrant location was considered as an independent risk factor for prognosis in breast cancer patients (15–17). It's well-recognized that axillary lymph node (ALN) metastasis is of predictive clinical significance for survival of breast cancer. And the possibility of ALN metastasis might be related to the primary location of tumor. Bevilacqua et al. suggested that the incidence of ALN metastasis is 32.3% when tumor is in central position, higher than tumor of upper-inner-quadrant (20.6%) (18). Japanese researchers investigated a cohort of 313 cases and found that T1 and T2 breast cancer patients with a tumor located closer to the nipple have a higher risk of sentinel lymph node (SLN) metastases (19). However, with regard to the effect of tumors located in central and nipple portion (TCNP) in Chinese breast cancer patients, few studies have been reported.

The purpose of this study was to investigate the clinicopathological characteristics of TCNP and to uncover its prognosis value in Chinese patients.

## Patients and methods

### Study population

The clinical and pathological information of 1,517 breast cancer patients who admitted to our department from December 2014 to December 2018 were collected. In this study, TCNP was defined as the tumor whose center was within 2 cm of the nipple, including the nipple-areola complex. We choose a 2 cm margin because it has been indicated that tumors within this zone involve the nipple-areolar complex in up to 50% of patients (20, 21). Tumors in the breast peripheral quadrant were defined as TBPQ (including upper outer quadrant, upper inner quadrant, lower outer quadrant, lower inner quadrant, but overlapping sites were excepted). The inclusion criteria were as follows: (1) females aged over 18 years old; (2) breast cancer as the first and only malignant primary tumor; (3) unilateral breast cancer; (4) defined tumour location (TCNP or TBPQ); (5) tumors must located in the same quadrant; (6) American Joint Committee on Cancer stages (the seventh AJCC System) TNM stage I–III; (7) breast molecular subtype (luminal A, luminal B, HER2 enriched, and triple-negative) (22). (8) known of estrogen receptor (ER); progesterone receptor (PR); HER2 expression; ki67; histological grade; tumor size and LN metastasis status; surgery type; radiation/chemotherapy information; (9) active follow-up. The exclusion criteria were: (1) male; (2) bilateral breast cancer; (3) occult breast cancer; (4) tumors were not clearly located and (or) involved two or more quadrants; (5) stage IV breast cancer at diagnosis; (6) patients who received neoadjuvant chemotherapy; (7) people with incomplete clinical or pathological information. According to the inclusion and exclusion criteria, 1,427 cases of breast cancer were finally enrolled, including 328 cases of TCNP and 1,099 cases of TBPQ. All patients have signed informed consent.

### Statistical analysis

Disease-free survival (DFS) was the primary end point. Overall survival (OS) and breast cancer-specific survival (BCSS) were the secondary end points. DFS was defined as the time from date of diagnosis to local or regional recurrence, distant organ metastasis, contralateral breast cancer, death or last follow-up. OS was defined as the time from diagnosis to death or last follow-up. BCSS was defined as the time from diagnosis to the death caused by breast cancer. Chi-square test was used to compare the clinical and pathological factors between TCNP and TBPQ groups. A one-to-one propensity score matching (PSM) analysis was carried out to balance the differences in baseline covariates, and we set the match tolerance as 0.02. Kaplan–Meier curves were applied to measure the DFS, BCSS, and OS between TCNP and TBPQ, and the differences were determined by log-rank test. Cox proportional hazard models were used to estimate risk ratios for prognostic factors. Logistic regression was applied to present the relationship between tumor location and LN metastasis. Two-sided *p* < 0.05 was considered statistically significant. All statistical analysis was completed by SPSS 26.0.

## Results

### Clinicopathological features of TCNP and TBPQ

As mentioned in above, a total of 1,427 breast cancer patients were eventually enrolled in this study with a median follow-up time of 58 months. The results before PSM indicated that compared to the TBPQ group, patients in the TCNP group had significantly larger tumor size (43.9% vs. 40.0% for tumor size of 2–5 cm; 7.6% vs. 3.0% for tumor size of >5 cm, *p* < 0.001), higher BMI (overweight and obese group: 42.7% vs. 35.9%, *p* = 0.037), higher rate of SLN metastasis (32.6% vs. 25.8%, *p* = 0.016), more frequent LN metastasis (1–3: 21.6% vs. 19.9%; ≥4: 23.8% vs. 13.2%, *p* < 0.001) more intravascular tumor thrombus (31.4% vs. 17.7%, *p* < 0.001) and more patients with advanced stages (TNM stage II–III) (69.2% vs. 58.2%, *p* < 0.001). The results also showed that more TCNP patients underwent mastectomy (89.9% vs. 59.3%, *p* < 0.001) but less of them received radiotherapy (34.8% vs. 45.9%, *p* < 0.001) (Table 1). These results reminded us that TCNP has unique clinical features which differ from TBPQ.

**Table 1:** Clinicopathological characteristics of TCNP and TBPQ groups.

Variables	Data before PSM *N* (%)		*p*-value	Data after PSM *N* (%)		*p*-value
	TCNP 328 (23.0)	TBPQ 1099 (77.0)		TCNP 326 (50)	TBPQ 326 (50)	
**Age (years)**			0.984			0.107
<50	136 (41.5)	455 (41.4)		134 (41.1)	114 (35.0)	
≥50	192 (58.5)	644 (58.6)		192 (58.9)	212 (65.0)	
**BMI (kg/m^2^)**			0.037*			0.107
Light: <18.5	23 (7.0)	55 (5.0)		23 (7.1)	14 (4.3)	
Normal: 18.5–23.9	165 (50.3)	650 (59.1)		163 (50.0)	192 (58.9)	
Overweight: 24–27.9	113 (34.5)	315 (28.7)		113 (34.7)	97 (29.8)	
Obesity: ≥28	27 (8.2)	79 (7.2)		27 (8.2)	23 (7.0)	
**Menopause**			0.599			0.178
Postmenopausal	180 (54.9)	585 (53.2)		180 (55.2)	197 (60.4)	
Premenopausal	148 (45.1)	514 (46.8)		146 (44.8)	129 (39.6)	
**Laterality**			0.883			0.309
Left	168 (51.2)	568 (51.7)		168 (51.5)	155 (47.5)	
Right	160 (48.8)	531 (48.3)		158 (48.5)	171 (52.5)	
**Surgery**			<0.001*			0.798
Lumpectomy	33 (10.1)	447 (40.7)		33 (10.1)	35 (10.7)	
Mastectomy	295 (89.9)	652 (59.3)		293 (89.9)	291 (89.3)	
**LN metastasis**			<0.001*			0.847
0	179 (54.6)	735 (66.9)		179 (54.9)	186 (57.1)	
1–3	71 (21.6)	219 (19.9)		70 (21.5)	68 (20.9)	
≥4	78 (23.8)	146 (13.2)		77 (23.6)	72 (22.0)	
**Tumor size (cm)**			<0.001*			0.366
≤2	159 (48.5)	626 (57.0)		159 (48.8)	144 (44.2)	
2–5	144 (43.9)	440 (40.0)		143 (43.9)	161 (49.4)	
>5	25 (7.6)	33 (3.0)		24 (7.3)	21 (6.4)	
**Intravascular tumor thrombus**			<0.001*			0.116
Yes	103 (31.4)	195 (17.7)		101 (31.0)	120 (36.8)	
No	225 (68.6)	904 (82.3)		225 (69.0)	206 (63.2)	
**Histological grade**			0.295			0.087
1	49 (14.9)	205 (18.7)		49 (15.0)	34 (10.5)	
2	204 (62.2)	647 (58.9)		204 (62.6)	229 (70.2)	
3	75 (22.9)	247 (22.4)		73 (22.4)	63 (19.3)	
**TNM stage**			<0.001*			0.800
I	101 (30.8)	459 (41.8)		101 (31.0)	104 (32.0)	
II–III	227 (69.2)	640 (58.2)		225 (69.0)	222 (68.0)	
**SLN metastasis**			0.016*			0.613
Yes	107 (32.6)	284 (25.8)		106 (32.5)	100 (30.7)	
No	221 (67.4)	815 (74.2)		220 (67.5)	226 (69.3)	
**ER**			0.570			0.867
Positive	222 (67.7)	762 (69.3)		220 (67.5)	222 (68.1)	
Negative	106 (32.3)	337 (30.7)		106 (32.5)	104 (31.9)	
**PR**			0.863			0.745
Positive	206 (62.8)	696 (63.3)		205 (62.9)	209 (64.1)	
Negative	122 (37.2)	403 (36.7)		121 (37.1)	117 (35.9)	
**Tumor subtype**			0.416			0.860
Luminal A	65 (19.8)	230 (20.9)		64 (19.6)	65 (19.9)	
Luminal B	162 (49.4)	567 (51.6)		162 (49.7)	168 (51.5)	
HER2+	55 (16.8)	144 (13.1)		55 (16.9)	47 (14.5)	
Triple-negative	46 (14.0)	158 (14.4)		45 (13.8)	46 (14.1)	
**Histology**			0.063			0.619
IDC	197 (60.1)	719 (65.4)		199 (61.0)	196 (60.2)	
ILC	6 (1.8)	26 (2.4)		11 (3.4)	6 (1.8)	
CIS	47 (14.3)	126 (11.5)		45 (13.8)	47 (14.4)	
Others	78 (23.8)	228 (20.7)		71 (21.8)	77 (23.6)	
**Radiotherapy**			<0.001*			0.678
Yes	114 (34.8)	504 (45.9)		112 (34.4)	107 (32.8)	
No	214 (65.2)	595 (54.1)		214 (65.6)	219 (67.2)	
**Chemotherapy**			0.251			0.860
Yes	241 (73.5)	779 (70.9)		239 (73.3)	237 (72.7)	
No	87 (26.5)	320 (29.1)		87 (26.7)	89 (27.3)	

Statistical significance was tested using chi-square test. TCNP, tumors located in central and nipple portions; TBPQ, tumors in the breast peripheral quadrant; PSM, propensity score matching; BMI, body mass index; SLN, sentinel lymph node; LN, lymph nodes; HER2, human epidermal growthfactor receptor 2; ER, estrogen receptor; PR, progesterone receptor; IDC, invasive ductal carcinoma; ILC, invasive lobular carcinoma; CIS, carcinoma *in situ*.

* Statistically significant.

After 1:1 matching, 326 patients in the TCNP group were matched as compared with 326 patients in the TBPQ group. There were no notable differences between the two groups after PSM (Table 1).

### Survival analysis between TCNP and TBPQ

**Figure 1:**
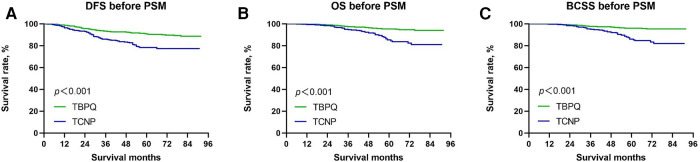
Kaplan–Meier survival curves of DFS (**A**), OS (**B**) and BCSS (**C**) between TCNP and TBPQ before PSM. DFS, disease-free survival; BCSS, breast cancer speciﬁc survival; OS, overall survival; PSM, propensity score matching.

**Figure 2:**
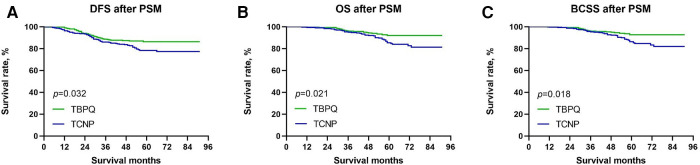
Kaplan–Meier survival curves of DFS (**A**), OS (**B**) and BCSS (**C**) between TCNP and TBPQ after PSM. DFS, disease-free survival; BCSS, breast cancer speciﬁc survival; OS, overall survival; PSM, propensity score matching.

The Kaplan–Meier curves were used to assess the differences of DFS, BCSS and OS between two groups. Before PSM, the 5-year DFS rates of patients with TCNP were significantly lower than those of TBPQ population (78.3% vs. 90.7%, *p* < 0.001) (Figure 1A). Next, we discovered that the 5-years OS rates of patients in the TCNP group were significantly worse than those of patients in the TBPQ group (82.4% vs. 94.5%, *p* < 0.001) (Figure 1B). Such difference was also observed in BCSS between TCNP and TBPQ (83.3% vs. 95.4%, *p* < 0.001) (Figure 1C). Similarly, we still found the notable difference between the two groups after PSM. The survival analysis of the matched groups showed that TCNP exhibited worse outcomes for DFS (5-year DFS: 78.5% vs. 86.3%, *p* = 0.032) (Figure 2A), OS (5-year OS: 85.4% vs. 92.1%, *p* = 0.0215) (Figure 2B) and BCSS (5-year BCSS: 86.2% vs. 92.8%, *p* = 0.018) (Figure 2C).

Furthermore, the Cox proportional hazards regression model were used to identify risk factors of DFS, OS and BCSS. Univariable Cox regression analysis presented that TCNP or TBPQ location, tumor size, intravascular tumor thrombus, TNM stage, LN metastasis, ER, PR, molecular subtype, chemotherapy, radiotherapy and histology were all associated with DFS. The significant predictors of OS were TCNP or TBPQ location, tumor size, TNM stage, LN metastasis, ER, PR and molecular subtype. Similarly, TCNP or TBPQ location, tumor size, TNM stage, LN metastasis, ER, PR, molecular subtype and chemotherapy were responsible for BCSS (Table 2). To balance the effect of factors, we further included the covariates that were clinically worth exploring or had *p* < 0.05 in the univariate Cox analysis into the multivariate Cox analysis. The results suggested that TCNP or TBPQ location, tumor size and LN metastasis were independent predictors of DFS (all *p* < 0.05), while menopause, TCNP or TBPQ location, tumor size, LN metastasis and PR were significant independent predictors of OS (all *p* < 0.05). Also, we found menopause, TCNP or TBPQ location, tumor size and LN metastasis were visibly associated with BCSS (all *p* < 0.05). TCNP was finally validated to be an independent risk factor for breast cancer prognosis (DFS: hazard ratios = 1.505, 95% CI: 1.008–2.246, *p* = 0.045; OS: hazard ratios = 2.038, 95% CI: 1.188–3.497, *p* = 0.010; BCSS: hazard ratios = 2.090, 95% CI: 1.187–3.680, *p* = 0.011) (Table 3).

**Table 2:** Univariate Cox analysis of DFS, OS and BCSS between TCNP and TBPQ.

Variables	DFS			OS			BCSS		
	HR	95% CI	*p*	HR	95% CI	*p*	HR	95% CI	*p*
**Age (years)**									
<50	Reference			Reference			Reference		
≥50	1.159	0.777–1.728	0.469	1.120	0.666–1.884	0.670	1.010	0.594–1.717	0.971
**Menopause**									
Postmenopausal	Reference			Reference			Reference		
Premenopausal	0.740	0.498–1.101	0.138	0.685	0.405–1.159	0.158	0.757	0.443–1.294	0.309
**Location**									
TBPQ	Reference			Reference			Reference		
TCNP	1.525	1.034–2.247	0.033*	1.836	1.089–3.097	0.023*	1.910	1.109–3.291	0.020*
**Surgery**									
Lumpectomy	Reference			Reference			Reference		
Mastectomy	1.531	0.744–3.148	0.247	1.497	0.599–3.740	0.388	1.389	0.554–3.481	0.483
**Tumor size (cm)**									
≤2	Reference			Reference			Reference		
2–5	1.323	0.876–1.999	0.183	1.430	0.821–2.488	0.206	1.359	0.766–2.413	0.295
>5	2.570	1.398–4.742	0.002*	3.412	1.606–7.248	0.001*	3.595	1.682–7.684	0.001*
**Intravascular tumor thrombus**									
No	Reference			Reference			Reference		
Yes	1.913	1.307–2.801	0.001*	1.518	0.919–2.508	0.103	1.401	0.832–2.358	0.205
**Histological grade**									
1	Reference			Reference			Reference		
2	1.508	0.753–3.020	0.247	1.439	0.566–3.656	0.444	1.365	0.535–3.477	0.515
3	2.105	0.993–4.462	0.052	2.681	1.000–7.188	0.050	2.391	0.881–6.489	0.087
**TNM stage**									
I	Reference			Reference			Reference		
II–III	2.961	1.715–5.113	<0.001*	2.317	1.178–4.561	0.015*	2.431	1.194–4.950	0.014*
**LN metastasis**									
0	Reference			Reference			Reference		
1–3	2.889	1.686–4.950	<0.001*	2.367	1.142–4.906	0.020*	2.354	1.106–5.010	0.026*
≥4	5.866	3.668–9.383	<0.001*	5.448	2.956–10.041	<0.001*	5.475	2.910–10.304	<0.001*
**ER**									
Positive	Reference			Reference			Reference		
Negative	1.662	1.131–2.443	0.010*	1.960	1.190–3.229	0.008*	2.084	1.245–3.488	0.005*
**PR**									
Positive	Reference			Reference			Reference		
Negative	1.593	1.087–2.333	0.017*	2.397	1.451–3.960	0.001*	2.442	1.452–4.107	0.001*
**Molecular subtype**									
Luminal A	Reference			Reference			Reference		
Luminal B	1.160	0.658–2.046	0.607	1.521	0.662–3.494	0.323	1.644	0.674–4.009	0.274
HER2+	1.882	0.988–3.583	0.054	2.902	1.183–7.122	0.020*	3.165	1.215–8.240	0.018*
Triple-negative	2.029	1.059–3.889	0.033*	2.863	1.142–7.177	0.025*	3.345	1.271–8.802	0.014*
**Chemotherapy**									
Yes	Reference			Reference			Reference		
No	0.359	0.201–0.641	0.001*	0.606	0.316–1.162	0.131	0.386	0.175–0.850	0.018*
**Radiotherapy**									
Yes	Reference			Reference			Reference		
No	0.544	0.371–0.797	0.002*	0.753	0.454–1.248	0.271	0.670	0.399–1.125	0.130
**Histology**									
IDC	Reference			Reference			Reference		
ILC	1.186	0.434–3.244	0.740	0.509	0.070–3.693	0.504	0.546	0.075–3.968	0.550
CIS	0.389	0.179–0.844	0.017*	0.395	0.142–1.099	0.075	0.423	0.152–1.178	0.100
Others	0.788	0.485–1.280	0.335	0.713	0.369–1.377	0.313	0.693	0.348–1.380	0.297

Statistical significance was tested using the Cox proportional hazards regression model. DFS, disease-free survival; BCSS, breast cancer-specific survival; OS, overall survival; HR, hazards ratios; CI, confidence interval; TCNP, tumors located in central and nipple portions; TBPQ, tumors in the breast peripheral quadrant; PSM, propensity score matching; LN, lymph nodes; HER2, human epidermal growthfactor receptor 2; ER, estrogen receptor; PR, progesterone receptor; IDC, invasive ductal carcinoma; ILC, invasive lobular carcinoma; CIS, carcinoma *in situ*.

* Statistically significant.

**Table 3:** Multivariate Cox analyses of DFS, OS and BCSS between TCNP and TBPQ.

Variables	DFS			OS			BCSS		
	HR	95% CI	*p*	HR	95% CI	*p*	HR	95% CI	*p*
**Age (years)**									
<50	Reference			Reference			Reference		
≥50	0.652	0.274–1.551	0.333	0.362	0.113–1.154	0.086	0.379	0.117–1.231	0.106
**Menopause**									
Postmenopausal	Reference			Reference			Reference		
Premenopausal	0.470	0.200–1.107	0.084	0.269	0.084–0.857	0.026*	0.304	0.094–0.986	0.047*
**Location**									
TBPQ	Reference			Reference			Reference		
TCNP	1.505	1.008–2.246	0.045*	2.038	1.188–3.497	0.010*	2.090	1.187–3.680	0.011*
**Surgery**									
Lumpectomy	Reference			Reference			Reference		
Mastectomy	0.738	0.342–1.595	0.440	0.661	0.244–1.790	0.415	0.634	0.232–1.730	0.374
**Tumor size (cm)**									
≤2	Reference			Reference			Reference		
2–5	0.944	0.571–1.560	0.822	1.327	0.643–2.738	0.444	1.245	0.598–2.593	0.558
>5	2.092	1.050–4.164	0.036*	3.877	1.573–9.555	0.003*	3.779	1.525–9.362	0.004*
**Histological grade**									
1	Reference			Reference			Reference		
2	1.177	0.573–2.418	0.657	1.182	0.449–3.111	0.735	1.085	0.408–2.883	0.870
3	1.041	0.474–2.288	0.920	1.323	0.469–3.731	0.596	1.096	0.386–3.114	0.864
**TNM stage**									
I	Reference			Reference			Reference		
II–III	0.754	0.305–1.863	0.541	0.317	0.089–1.127	0.076	0.360	0.099–1.310	0.121
**LN metastasis**									
0	Reference			Reference			Reference		
1–3	3.378	1.617–7.057	0.001*	4.846	1.671–14.052	0.004*	3.943	1.349–11.526	0.012*
≥4	6.919	3.496–13.693	<0.001*	11.659	4.334–31.366	<0.001*	9.638	3.572–26.004	<0.001*
**ER**									
Positive	Reference			Reference			Reference		
Negative	1.304	0.383–4.443	0.671	1.316	0.220–7.887	0.763	1.418	0.237–8.473	0.702
**PR**									
Positive	Reference			Reference			Reference		
Negative	1.067	0.479–2.378	0.874	2.544	1.106–5.851	0.028*	2.407	0.990–5.847	0.053
**Molecular subtype**									
Luminal A	Reference			Reference			Reference		
Luminal B	0.862	0.480–1.547	0.618	1.143	0.483–2.702	0.761	1.169	0.465–2.938	0.740
HER2+	1.014	0.218–4.713	0.986	0.734	0.087–6.197	0.776	0.746	0.086–6.503	0.791
Triple-negative	1.300	0.286–5.915	0.734	0.865	0.104–7.184	0.893	0.975	0.114–8.318	0.981
**Chemotherapy**									
Yes	Reference			Reference			Reference		
No	0.757	0.396–1.447	0.400	1.507	0.719–3.162	0.278	0.888	0.371–2.127	0.791

Statistical significance was tested using the Cox proportional hazards regression model. DFS, disease-free survival; BCSS, breast cancer-specific survival; OS, overall survival; HR, hazards ratios; CI, confidence interval; TCNP, tumors located in central and nipple portions; TBPQ, tumors in the breast peripheral quadrant; PSM, propensity score matching; LN, lymph nodes; HER2, human epidermal growthfactor receptor 2; ER, estrogen receptor; PR, progesterone receptor.

* Statistically significant.

### Subgroup analysis

**Figure 3:**
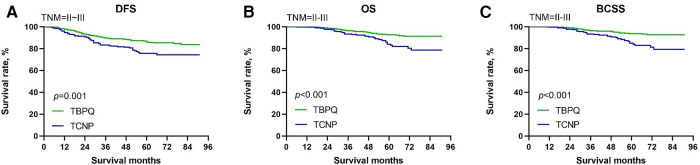
Kaplan–Meier survival curves of DFS (**A**), OS (**B**) and BCSS (**C**) between TCNP and TBPQ in TNM stage II–III subgroup. DFS, disease-free survival; BCSS, breast cancer speciﬁc survival; OS, overall survival.

**Figure 4:**
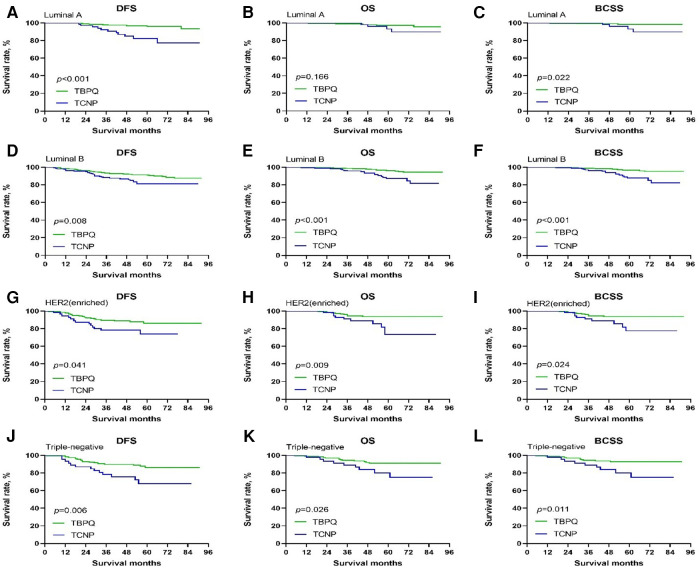
Kaplan–Meier survival curves of DFS, OS and BCSS between TCNP and TBPQ in different breast cancer molecular subtypes. DFS (**A**), OS (**B**) or BCSS (**C**) between TCNP and TBPQ in luminal A breast cancer. DFS (**D**), OS (**E**) or BCSS (**F**) between TCNP and TBPQ in luminal B breast cancer. DFS (**G**), OS (**H**) or BCSS (**I**) between TCNP and TBPQ in HER2 enriched breast cancer. DFS (**J**), OS (**K**) or BCSS (**L**) between TCNP and TBPQ in triple-negative breast cancer. DFS, disease-free survival; BCSS, breast cancer speciﬁc survival; OS, overall survival.

Now that TCNP was verified to be significantly negatively correlated with DFS, OS and BCSS of breast cancer, we were interested to further evaluated the potential prognostic value of TCNP in subgroups. Patients in TCNP or TBPQ were further stratified based on important clinical features. For patient with TNM stage II–III, we found that TCNP was a worse prognostic indicator for DFS (*p* = 0.001), OS (*p* < 0.001) and BCSS (*p* < 0.001) (Figure 3). Noticeable difference could also be seen in subgroups of breast molecular subtypes. For luminal B, HER2 enriched and triple-negative breast cancer, TCNP patients had decreased DFS, OS and BCSS as compared to TBPQ patients (all *p* < 0.05) (Figures 4D–L). Although prognostic value of TCNP for OS in luminal A patients had no significance (*p* = 0.166) (Figure 4B), TCNP still gave visible prognostic value for predicting poorer DFS (*p* < 0.001) and BCSS (*p* = 0.022) in luminal A subgroup (Figures 4A,C). Furthermore, we performed a detailed analysis on prognosis of lateral and medial breast cancers. There were 681 patients with tumors in the lateral quadrant and 418 patients with tumors in the medial quadrant. Compared to the medial group, the group of lateral location were older 62.1% vs. 52.9% for age ≥50 years, *p* = 0.003) and had more proportion of postmenopausal patients (57.0% vs. 47.1%, *p* = 0.001), larger tumor size (45.7% vs. 38.7% for tumor size of >2 cm, *p* = 0.048), more SLN (28.0% vs. 22.2%, *p* = 0.033) and LN metastases (≥4: 15.4% vs. 9.6%, *p* = 0.019). Besides, TNM stage was more advanced in lateral group (61.1% vs. 53.6% for TNM stage II–III, *p* = 0.014). Survival analysis demonstrated that no significant differences were seen for DFS, OS and BCSS between the two groups before and after PSM (all *p* > 0.05) (see Supplementary Material for details).

### TCNP and LN metastasis

In addition, we analyzed the correlation between unfavorable prognosis of TCNP patients and LN metastasis. Univariate logistic analysis was performed and significant (*p* < 0.05) variables (TCNP or TBPQ location, tumor size, histological grade, TNM stage, molecular subtype and histology) were further incorporated into the multivariate logistic regression analysis. TCNP was finally proved to be more susceptible to LN metastasis than TBPQ (OR = 1.820, 95% CI: 1.251–2.649, *p* = 0.002) (Table 4).

**Table 4:** Univariate and multivariate logistic regression analysis on LN metastasis.

Characteristics	Univariate analysis			Multivariate analysis		
	OR	95% CI	*p*-value	OR	95% CI	*p*-value
**Age (years)**						
<50	Reference			Reference		
≥50	1.098	0.881–1.369	0.430	1.104	0.604–2.019	0.748
**Menopause**						
Postmenopausal	Reference			Reference		
Premenopausal	0.842	0.678–1.047	0.122	1.346	0.748–2.423	0.321
**Location**						
TBPQ	Reference			Reference		
TCNP	1.681	1.308–2.160	<0.001*	1.820	1.251–2.649	0.002*
**Tumor size (cm)**						
≤2	Reference			Reference		
2–5	2.055	1.642–2.572	<0.001*	0.019	0.007–0.048	<0.001*
>5	1.512	0.870–2.626	0.143	0.014	0.005–0.042	<0.001*
**Histological grade**						
1	Reference			Reference		
2	2.654	1.878–3.750	<0.001*	2.264	1.220–4.202	0.010*
3	3.654	2.485–5.371	<0.001*	2.236	1.146–4.363	0.018*
**TNM stage**						
I	Reference			Reference		
II–III	157.070	64.452–382.780	<0.001*	4564.176	1286.528–16192.185	<0.001*
**Molecular subtype**						
Luminal A	Reference			Reference		
Luminal B	1.905	1.409–2.574	<0.001*	1.275	0.744–2.185	0.377
HER2+	1.972	1.341–2.901	0.001*	1.314	0.689–2.507	0.407
Triple-negative	1.566	1.061–2.312	0.024	1.174	0.620–2.223	0.622
**Histology**						
IDC	Reference			Reference		
ILC	0.848	0.414–1.738	0.653	1.277	0.453–3.602	0.643
CIS	0.037	0.015–0.091	<0.001*	0.049	0.018–0.132	<0.001*
Others	0.485	0.366–0.642	<0.001*	0.477	0.319–0.714	<0.001*

Statistical significance was tested using logistic regression. LN, lymph nodes; OR, odds ratio; CI, confidence interval; TCNP, tumors located in central and nipple portions; TBPQ, tumors in the breast peripheral quadrant; HER2, human epidermal growthfactor receptor 2; IDC, invasive ductal carcinoma; ILC, invasive lobular carcinoma; CIS, carcinoma *in situ*.

* Statistically significant.

## Discussion

Although several studies have shown that the location of the primary tumor has an important effect on prognosis, it has not been well adopted in clinic as a prognostic risk factor (23–28). Therefore it is critical to better understand the association between location of the primary tumor and its influence on the disease outcome of breast cancer in order to develop specific treatment in the future. TCNP is a relatively unique site, and has been rarely studied in the field, especially in Asian populations. In this retrospective study of 1,427 cases, we uncovered that among Chinese population, TCNP presents distinct clinicopathological features and worse prognosis than TBPQ.

First of all, our results showed that compared to patients in TBPQ group, patients from TCNP group were accompanied with larger tumor sizes (>2 cm), higher BMI, higher rates of LN metastasis, more advanced TNM stages (II-III) and more intravascular tumor thrombus. These unfavorable clinical characteristics of TCNP might contribute to its larger tumor burden and worse survival outcomes than TBPQ. On the one hand, TCNP patients had higher BMI and more adipose tissue especially in the breast might increase the difficulty for early detection of the mass when undergo the B-ultrasonic examination; on the other hand, during mammography, excessive x-ray penetration in the nipple-areola complex reduced the accuracy of the examination (29).

The prognostic value of primary tumor site is currently highly debated and remains unclear. Siotos et al. suggested that the site of the primary tumor might be an important feature affecting the prognosis of breast cancer in a whole cohort of 5,295 patients (10). Rummel et al. reported that although tumours in the central region were associated with less favourable outcome, these associations were not independent of location but rather driven by larger tumour size (30). Wu et al. found that in Chinese women with T1-2N0M0 breast cancer, the inner and lower quadrant was an independent risk factor for DFS and OS, while tumor in the central region had no prognostic value (31). Another study showed that in invasive ductal breast carcinoma, patients with tumors in the central and medial quadrants had significantly increased risk of recurrence, metastasis, and death compared to patients with tumors in other quadrants (32). However, in our study, the prognosis for lateral breast cancer was similar to that for medial breast cancer, which was consistent with the previous study by Jayasinghe et al. (33). Many factors, for example, LN metastasis, TCNP or TBPQ location, tumor size, TNM stage, ER, PR and molecular subtype were associated with DFS, OS and BCSS in univariate analysis. After multivariate analysis, TCNP was substantiated as an independent risk predictor over TBPQ for both DFS, OS and BCSS. The survival analysis revealed that among Chinese female patients, the DFS, BCSS and OS rates of TCNP were significantly lower than those of TBPQ. The findings of our research support another recent investigation which suggested that central breast cancer has poorer BCSS and OS than non-central breast cancer based on population from SEER database (34).

Further subgroup analysis demonstrated that TCNP was a poor prognostic indicator of DFS, OS and BCSS in Chinese patients with luminal B, HER2 enriched, triple-negative and TNM stage II–III breast cancer. As for luminal A subtype, TCNP still had power of predicting worse DFS and BCSS. These findings strongly supported that TCNP served as a robust indicator of poor prognosis among Chinese breast cancer patients.

The mechanism underlying poor prognosis for TCNP may be as follows. Firstly, tumors of TCNP are easily missed and delayed in diagnosis as mentioned above, which can affect treatment. Secondly, higher rate of ALN metastasis in TCNP is another leading cause for decreased survival. The number of ALN that metastases of breast cancer is of predictive clinical significance (35) and Involvement of ALN has been believed to be accountable for increasing breast cancer recurrence and mortality (36, 37). Those who initially presented with ALN metastases usually received worse survival after recurrence (38, 39). To date, a clear association between LN metastasis and lumps located near the nipple and areola has not been establish, yet many scholars hold a supportive attitude. A landmark study by Ansari et al. demonstrated that for every 1 cm decrease in the distance between the tumor and the nipple, the likelihood of LN positivity increases by 23% (40). A recent study by Yang et al. revealed that tumour-nipple distance was an independent predictor of ALN involvement. In LN-positive patients, the tumour-nipple distance was smaller (41). In this study, TCNP was found to have a higher proportion of LN metastasis than TBPQ. Through further univariate and multivariate logistic regression analysis, we finally elucidated that TCNP was an independent indicator for LN metastasis. Our result is in corroboration with the previous findings. Last but not least, the role of internal mammary lymph node (IMLN) in TCNP cannot be overlooked. IMLN is the second largest lymphatic drainage of breast cancer, after the ALN (42). It is generally believed that medial and central tumors more often drain to the IMLN than other quadrants and IMLN metastasis is always found concomitantly with ALN metastasis. A large sample retrospective study by Huang et al. indicated that the incidence of IMLN metastasis was 4.4%, 18.8%, 28.1%, and 41.5% for patients with negative ALN, 1–3 positive ALN, 4–6 positive ALN, seven or more positive ALN, respectively (43). The status of IMLN is also an important factor for determining the clinical stage, treatment strategy and prognosis of breast cancer patients. Veronesi et al. retrospectively analyzed 1,085 patients and showed that patients with ALN metastases only or with IMLN metastases only had similar prognosis, while patients with both axillary and internal mammary positive nodes had the worst prognosis (44). Although our study did not evaluate IMLN due to the deep anatomical location, small size of IMLN, low accuracy and sensitivity of the current used tracer and no consensus on the indications for IMLN biopsy and dissection, we successfully proved TCNP more prone to ALN metastases (even in subgroup of metastatic LN number ≥4). Therefore, we could logically assume that IMLN increases the adverse impact of TCNP on prognosis.

Our study has several important strengths. Firstly, this is a unique study that focused on the potential clinical value of TCNP for Chinese population. In China, the incidence rate of breast cancer has soared obviously in recent decades and our results represent real-world data that may be generalisable to routine clinical settings. Secondly, the relatively large sample size and long duration of follow up are other strengths of this analysis. Our results indicated that for TCNP patients, clinicians need to pay more attention to assessing the status of LN and improve preoperative evaluation comprehensively. Also, we suggest that it's necessary to consider whether the primary tumor site should be included in breast cancer staging guidelines.

This study has some limitations that should be noted. First, although the data is real and effective, this is a retrospective study from a single center and a selection bias cannot be entirely excluded. Second, evaluation of IMLN are not performed, therefore, we are unable to determine whether IMLN metastasize. More studies involving prospective and multicenter data collection are needed to confirm the clinical predicting value of tumor location in breast cancer patients of Asian/Chinese origin.

Summarily, the current study indicated that TCNP is an independent prognostic factor for Chinese breast cancer, which is correlated with impaired survival and more likely to have LN metastasis. Our findings fill the important gap in the literature by discovering TCNP's role in Chinese breast cancer population. We suggest that prompt diagnosis and effective treatment are needed for TCNP patients in clinical practice.

## Data Availability

The original contributions presented in the study are included in the article/**Supplementary Material**, further inquiries can be directed to the corresponding author/s.
