# Supplementary material for: Real-world outcomes for Chinese breast cancer patients with tumor location of central and nipple portion
Source: Front Surg. 2022 Oct 3;9:993263. doi: 10.3389/fsurg.2022.993263 (PMC9574339; doi:10.3389/fsurg.2022.993263)
Supplement: Supplementary file 1 [file Table1.docx]

**Supplemental Table. Clinicopathological characteristics of Lateral and Medial groups**

| **Variables** | **Data before PSM *N* (%)** | | ***P*-value** | **Data after PSM *N* (%)** | | ***P*-value** |
| --- | --- | --- | --- | --- | --- | --- |
|  | **Lateral 681 (62.0)** | **Medial 418 (38.0)** |  | **Lateral 415 (50.0)** | **Medial 415 (50.0)** |  |
| **Age (years)** |  |  | 0.003^#^ |  |  | 0.728 |
| <50 | 258 (37.9) | 197 (47.1) |  | 189 (45.5) | 194 (46.7) |  |
| ≥50 | 423 (62.1) | 221 (52.9) |  | 226 (54.5) | 221 (53.3) |  |
| **BMI (kg/m^2^)** |  |  | 0.632 |  |  | 0.958 |
| Light:<18.5 | 34 (5.0) | 21 (5.0) |  | 23 (5.5) | 21 (5.1) |  |
| Normal:18.5-23.9 | 410 (60.2) | 240 (57.4) |  | 243 (58.5) | 238 (57.3) |  |
| Overweight:24-27.9 | 193 (28.3) | 122 (29.2) |  | 116 (28.0) | 122 (29.4) |  |
| Obesity:≥28 | 44 (6.5) | 35 (8.4) |  | 33 (8.0) | 34 (8.2) |  |
| **Menopause** |  |  | 0.001^#^ |  |  | 0.835 |
| Postmenopausal | 388 (57.0) | 197 (47.1) |  | 200 (48.2) | 197 (47.5) |  |
| Premenopausal | 293 (43.0) | 221 (52.9) |  | 215 (51.2) | 218 (52.5) |  |
| **Laterality** |  |  | 0.107 |  |  | 1.000 |
| Left | 339 (49.8) | 229 (54.8) |  | 227 (54.7) | 227 (54.7) |  |
| Right | 342 (50.2) | 189 (45.2) |  | 188 (45.3) | 188 (45.3) |  |
| **Surgery** |  |  | 0.898 |  |  | 0.888 |
| Lumpectomy | 278 (40.8) | 169 (40.4) |  | 171 (41.2) | 169 (40.7) |  |
| Mastectomy | 403 (59.2) | 249 (59.6) |  | 244 (58.8) | 246 (59.3) |  |
| **SLN metastasis** |  |  | 0.033^#^ |  |  | 0.741 |
| Yes | 191 (28.0) | 93 (22.2) |  | 97 (23.4) | 93 (22.4) |  |
| No | 490 (72.0) | 325 (77.8) |  | 318 (76.6) | 322 (77.6) |  |
| **LN metastasis** |  |  | 0.019^#^ |  |  | 0.300 |
| 0 | 441 (64.8) | 294 (70.3) |  | 295 (71.1) | 291 (70.2) |  |
| 1-3 | 135 (19.8) | 84 (20.1) |  | 70 (16.9) | 84 (20.2) |  |
| ≥4 | 105 (15.4) | 40 (9.6) |  | 50 (12.0) | 40 (9.6) |  |
| **Tumor size (cm)** |  |  | 0.048^#^ |  |  | 0.609 |
| ≤2 | 370 (54.3) | 256 (61.2) |  | 244 (58.8) | 253 (61.0) |  |
| 2-5 | 292 (42.9) | 148 (35.4) |  | 160 (38.5) | 148 (35.6) |  |
| >5 | 19 (2.8) | 14 (3.3) |  | 11 (2.7) | 14 (3.4) |  |
| **Intravascular tumor thrombus** |  |  | 0.766 |  |  | 1.000 |
| Yes | 119 (17.5) | 76 (18.2) |  | 75 (18.1) | 75 (18.1) |  |
| No | 562 (82.5) | 342 (81.8) |  | 340 (81.9) | 340 (81.9) |  |
| **Histological grade** |  |  | 0.509 |  |  | 0.695 |
| 1 | 120 (17.6) | 85 (20.3) |  | 79 (19.0) | 83 (20.0) |  |
| 2 | 404 (59.3) | 243 (58.1) |  | 249 (60.0) | 292 (70.4) |  |
| 3 | 157 (23.1) | 90 (21.5) |  | 87 (21.0) | 90 (21.7) |  |
| **TNM stage** |  |  | 0.014^#^ |  |  | 0.530 |
| I | 265 (38.9) | 194 (46.4) |  | 182 (43.9) | 191 (46.0) |  |
| II-III | 416 (61.1) | 224 (53.6) |  | 233 (56.1) | 224 (54.0) |  |
| **ER** |  |  | 0.041^#^ |  |  | 0.876 |
| Positive | 457 (67.1) | 305 (73.0) |  | 300 (72.3) | 302 (72.8) |  |
| Negative | 224 (32.9) | 113 (27.0) |  | 115 (27.7) | 113 (27.2) |  |
| **PR** |  |  | 0.049^#^ |  |  | 1.000 |
| Positive | 416 (61.1) | 280 (67.0) |  | 278 (67.0) | 278 (67.0) |  |
| Negative | 265 (38.9) | 138 (33.0) |  | 137 (33.0) | 137 (33.0) |  |
| **Tumor subtype** |  |  | 0.122 |  |  | 0.996 |
| Luminal A | 138 (20.2) | 92 (22.1) |  | 90 (21.7) | 92 (22.2) |  |
| Luminal B | 339 (49.8) | 228 (54.5) |  | 225 (54.2) | 225 (54.2) |  |
| HER2+ | 95 (14.0) | 49 (11.7) |  | 51 (12.3) | 49 (11.8) |  |
| Triple-negative | 109 (16.0) | 49 (11.7) |  | 49 (11.8) | 49 (11.8) |  |
| **Histology** |  |  | 0.857 |  |  | 0.936 |
| IDC | 441 (64.8) | 278 (66.5) |  | 279 (67.2) | 275 (66.2) |  |
| ILC | 17 (2.5) | 9 (2.2) |  | 9 (2.2) | 9 (2.2) |  |
| CIS | 82 (12.0) | 44 (10.5) |  | 47 (11.3) | 44 (10.6) |  |
| Others | 141 (20.7) | 87 (20.8) |  | 80 (19.3) | 87 (21.0) |  |
| **Radiotherapy** |  |  | 0.558 |  |  | 0.676 |
| Yes | 317 (46.5) | 187 (44.7) |  | 193 (46.5) | 187 (45.1) |  |
| No | 364 (53.5) | 231 (55.3) |  | 222 (53.5) | 228 (54.9) |  |
| **Chemotherapy** |  |  | 0.923 |  |  | 0.761 |
| Yes | 482 (70.8) | 297 (71.1) |  | 290 (69.9) | 294 (70.8) |  |
| No | 199 (29.2) | 121 (28.9) |  | 125 (30.1) | 121 (29.2) |  |

Statistical significance was tested using chi-square test.

^#^Statistically significant.

Abbreviations: propensity score matching=PSM; body mass index=BMI; Sentinel lymph node=SLN; lymph nodes=LN; human epidermal growthfactor receptor 2=HER2; estrogen receptor=ER; progesterone receptor=PR; invasive ductal carcinoma=IDC; invasive lobular carcinoma=ILC; carcinoma in situ=CIS.
