# Supplementary figures and images for: Real-world outcomes for Chinese breast cancer patients with tumor location of central and nipple portion
Source: Front Surg. 2022 Oct 3;9:993263. doi: 10.3389/fsurg.2022.993263 (PMC9574339; doi:10.3389/fsurg.2022.993263)

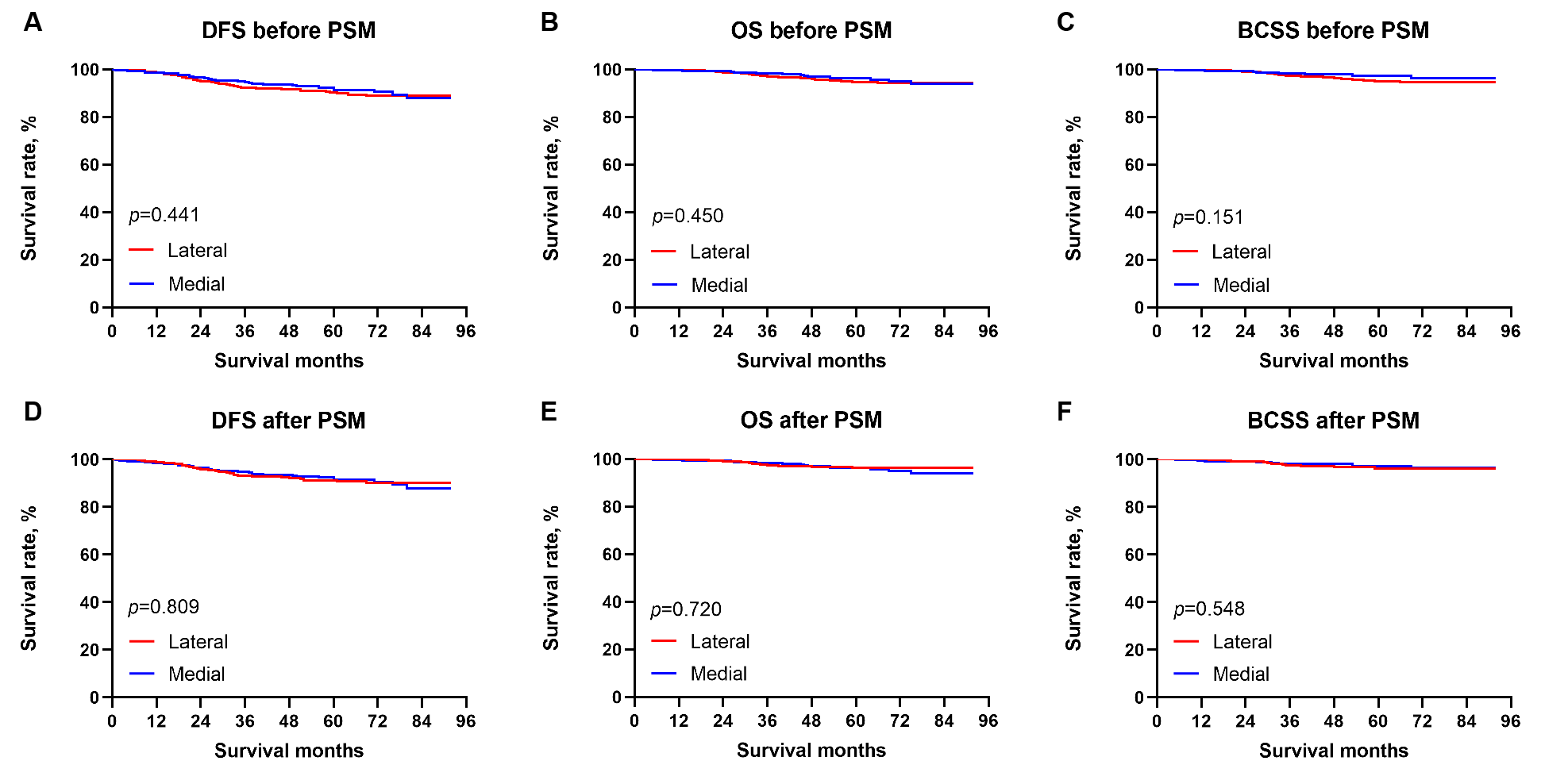

Supplement: Supplementary file 2 [file Image1.tif]
